# Supplementary material for: Partial Endothelial Nitric Oxide Synthase Deficiency Exacerbates Cognitive Deficit and Amyloid Pathology in the APPswe/PS1ΔE9 Mouse Model of Alzheimer’s Disease
Source: Int J Mol Sci. 2022 Jun 30;23(13):7316. doi: 10.3390/ijms23137316 (PMC9266765; doi:10.3390/ijms23137316)
Supplement: Supplementary file 1 [file ijms-23-07316-s001.zip › ijms-1798576-supplementary.pdf]

Supplementary Table S1:

Mean (± SEM) organ (mg)/body (g) ratios in 8 months old male wildtype (WT), $eNOS^{+/-}$, APP/PS1 and APP/PS1/$eNOS^{+/-}$ mice at the time of tissue collection (n = 8-12/genotype; presented as percentage of body weight).

Organ/body weight ratio	Brain	Forebrain	Cerebellum	Liver	Spleen	Heart	Kidney (L)	Kidney (R)
**WT**	12.95 ± 0.416	9.67 ± 0.334	1.873 ± 0.068	46.75 ± 1.200	2.443 ± 0.110	5.170 ± 0.206	5.529 ± 0.143	6.093 ± 0.184
**$eNOS^{+/-}$**	14.12 ± 0.473	10.46 ± 0.344	2.038 ± 0.083	46.09 ± 1.856	2.450 ± 0.143	4.928 ± 0.297	5.553 ± 0.344	6.043 ± 0.384
**APP/PS1**	13.93 ± 0.221	10.46 ± 0.185	1.919 ± 0.044	43.30 ± 1.270	2.398 ± 0.097	4.921 ± 0.170	6.075 ± 0.295	6.184 ± 0.203
**APP/PS1/$eNOS^{+/-}$**	14.23 ± 0.535	10.72 ± 0.392	2.044 ± 0.096	43.34 ± 1.033	2.128 ± 0.106	4.829 ± 0.252	5.751 ± 0.160	6.181± 0.222
**ANOVA results**	$F_{(3,37)} = 2.20$ $p = 0.11$	$F_{(3,37)} = 2.27$ $p = 0.10$	$F_{(3,37)} = 1.41$ $p = 0.25$	$F_{(3,37)} = 1.93$ $p = 0.14$	$F_{(3,37)} = 1.67$ $p = 0.19$	$F_{(3,37)} = 0.46$ $p = 0.72$	$F_{(3,37)} = 1.17$ $p = 0.33$	$F_{(3,37)} = 0.08$ $p = 0.97$

Abbreviations: L, left; R, right.
